# HLA-EpiCheck: novel approach for HLA B-cell epitope prediction using 3D-surface patch descriptors derived from molecular dynamic simulations

**DOI:** 10.1093/bioadv/vbae186

**Published:** 2024-12-05

**Authors:** Diego Amaya-Ramirez, Magali Devriese, Romain Lhotte, Cédric Usureau, Malika Smaïl-Tabbone, Jean-Luc Taupin, Marie-Dominique Devignes

**Affiliations:** LORIA, Université de Lorraine, CNRS, INRIA, Nancy 54000, France; Hôpital Saint-Louis, Inserm, Paris 75010, France; Hôpital Saint-Louis, Inserm, Paris 75010, France; Hôpital Saint-Louis, Inserm, Paris 75010, France; LORIA, Université de Lorraine, CNRS, INRIA, Nancy 54000, France; Hôpital Saint-Louis, Inserm, Paris 75010, France; LORIA, Université de Lorraine, CNRS, INRIA, Nancy 54000, France

## Abstract

**Motivation:**

The human leukocyte antigen (HLA) system is the main cause of organ transplant loss through the recognition of HLAs present on the graft by donor-specific antibodies raised by the recipient. It is therefore of key importance to identify all potentially immunogenic B-cell epitopes on HLAs in order to refine organ allocation. Such HLAs epitopes are currently characterized by the presence of polymorphic residues called “eplets”. However, many polymorphic positions in HLAs sequences are not yet experimentally confirmed as eplets associated with a HLA epitope. Moreover, structural studies of these epitopes only consider 3D static structures.

**Results:**

We present here a machine-learning approach for predicting HLA epitopes, based on 3D-surface patches and molecular dynamics simulations. A collection of 3D-surface patches labeled as Epitope (2117) or Nonepitope (4769) according to Human Leukocyte Antigen Eplet Registry information was derived from 207 HLAs (61 solved and 146 predicted structures). Descriptors derived from static and dynamic patch properties were computed and three tree-based models were trained on a reduced non-redundant dataset. HLA-Epicheck is the prediction system formed by the three models. It leverages dynamic descriptors of 3D-surface patches for more than half of its prediction performance. Epitope predictions on unconfirmed eplets (absent from the initial dataset) are compared with experimental results and notable consistency is found.

**Availability and implementation:**

Structural data and MD trajectories are deposited as open data under doi: 10.57745/GXZHH8. In-house scripts and machine-learning models for HLA-EpiCheck are available from https://gitlab.inria.fr/capsid.public_codes/hla-epicheck.

## 1 Introduction

The human leukocyte antigen (HLA) system plays a key role in activating the human immune response. It is responsible for presenting peptides to T-lymphocytes after antigen processing in cells. However, the same system is mainly responsible for the loss of organ transplants as the genes encoding HLA proteins are the most polymorphic human genes. Consequently, it is extremely difficult to find HLA-identical donors and recipients outside of twins, and therefore, transplantations are almost always performed across HLA polymorphisms that may later trigger an immune response (both humoral and cellular) against the donor. The humoral response occurs when recipient B-cells start to produce antibodies capable of recognizing the donor HLA antigens as foreign proteins. Produced antibodies are named “donor-specific antibodies” (DSA) and may lead to graft rejection. The prevention of humoral response in transplantation relies on (i) proper donor–recipient matching ahead of graft allocation and (ii) well-adjusted immmunosuppressive treatment after transplantation. For both purposes, it is interesting to know the mismatch load (MML) between donor and recipient HLA antigens, i.e. the sequence and structure differences that will make the donor proteins be recognized as foreign proteins by the recipient.

**Figure 1.**
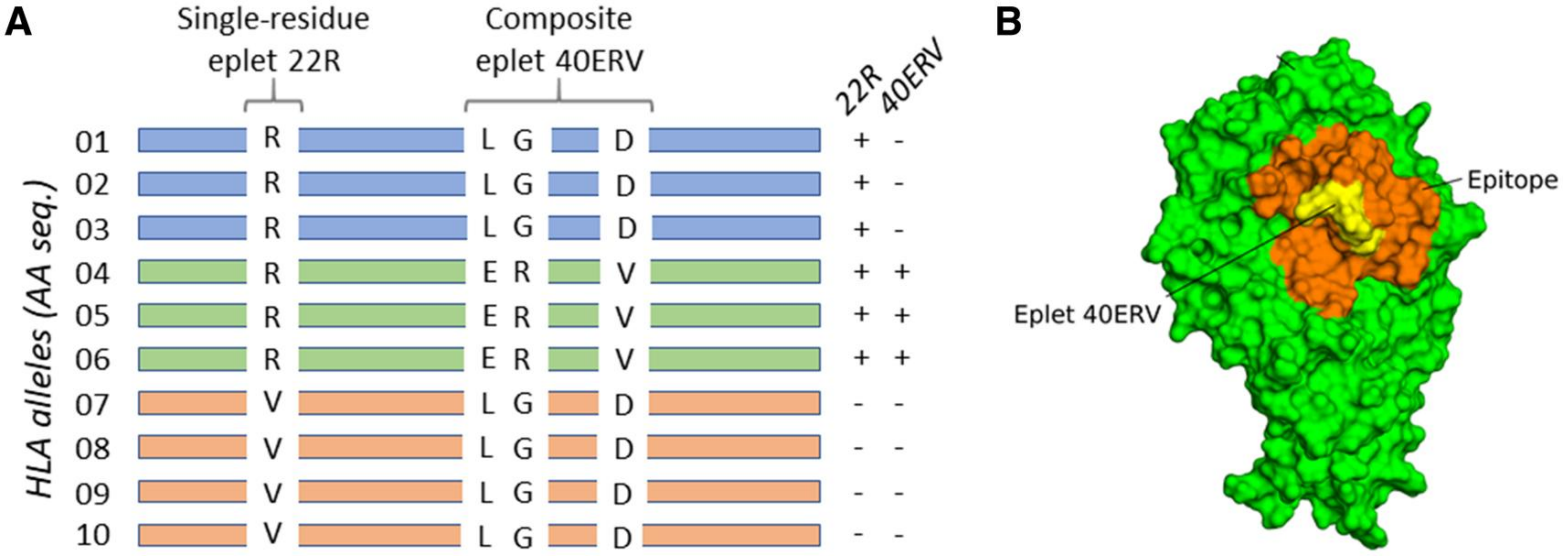
Schema explaining the concept of single-residue and composite eplets. (A). Sequence level. A fictitious set of 10 aligned HLA AA sequences (alleles of the same gene) is schematized. Fictitious differences in AA sequences are highlighted. Eplets are defined by a position in the HLA AA sequence and the nature of the residue that induces antibody recognition. Here, single-residue eplet 22R is present in sequences 01–06 but absent from the remaining sequences. Composite eplet 40ERV is composed of three non-consecutive AAs at positions 40, 41, and 45. This eplet is present in sequences 04–06 but absent from all other sequences. Sequences 07–10 do not carry any of these two eplets but may carry other eplets elsewhere in their sequence. (B). 3D-structure level. Surface representation of antigen HLA DQA1*02:01-DQB1*02:01. This structure is one of those produced in this study by AlphaFold-2 prediction. Real composite eplet 40ERV mapping at positions 40, 41, and 45 in the *α* chain of this antigen is shown in yellow. The orange zone corresponds to surface residues that are 15 Å at most away from the three residues of eplet 40ERV. It corresponds to the merging of the three 3D-surface patches built from this structure around residues 40E, 41R, and 45V. This zone represents a putative epitope.

HLA genes are localized on human chromosome 6, position 6p21.31 and are classified as class I for the A, B, and C loci and class II for the DR, DQ, and DP loci. Class II loci are more complex as DQ and DP antigens associate two genes that are both polymorphic (DQA1 and DQB1 for DQ, DPA1 and DPB1 for DP). Thousands of alleles have been described for each HLA gene with more than 90% sequence similarity between them for a given gene. Thus, HLA antigens are often qualified as “highly polymorphic in a context of high homology” (Tambur 2018). Indeed, two HLA antigens may differ by only one amino acid (AA), but this single difference can induce a significant change in their recognition by antibodies (Fig. 1).

A B-cell epitope covers a defined region of 600–900 ${Å}^{2}$ on protein surface, i.e. a pseudo-disk with a radius of about 15 Å, that is recognized and bound by antibodies produced by B-cells (Fig. 1). B-cell epitopes are present on all sorts of proteins, not only HLA antigens. This differs from T-cell epitopes which are specific for HLA antigens since they are formed by the presentation to T-cell receptors of antigenic peptides (derived from intracellular digestion of foreign proteins) in a structural groove present in all HLA antigens. The recognition of T-cell epitopes by T-cells initiates the cellular immune response. In this article, we focus on the 3D structure of B-cell epitopes on HLA antigens as they are ultimately responsible for DSA recognition and binding. The notation will be simplified here as “HLA epitope” as it is the only type of epitope considered in this study.

Initially, the task of estimating MML between recipient and donor was limited to counting the different polymorphic AA triplets between recipient’s and donor’s HLA antigens. The pioneer system in the field is certainly HLAMatchmaker (Duquesnoy 2002). This simple system was rapidly improved and gave rise to the notion of polymorphic eplets rather than triplets (Duquesnoy and Askar 2007). Eplets are discontinuous polymorphic residues located not more than 3–3.5 Å from each other in the 3D structure of HLA antigens. They constitute the key components of the allogeneic HLA epitopes recognized by DSA in humans (see Fig. 1). A well maintained database [HLA Eplet Registry (https://www.epregistry.com.br/)] stores and documents putative and antibody-verified eplets reported in the scientific literature (Duquesnoy *et al.* 2013). Antibody-verified eplets (also known as “confirmed” eplets) are those for which an experimental validation (e.g. with patients’ sera) has been performed, in contrast to putative eplets (also known as “unconfirmed” eplets) which are essentially deduced from alignments of HLA antigens sequences. Several programs and web servers [e.g. HLAMatchmaker (http://www.epitopes.net/downloads.html)] allow MML computation based on simple eplet counting (da Mata Sousa *et al.* 2011, Geffard *et al.* 2022). Despite various retrospective studies about possible correlation between antibody-verified eplet MML and DSA formation or graft survival (Wiebe *et al.* 2019, Sapir-Pichhadze *et al.* 2020, Senev *et al.* 2020), no agreement exists yet in favor of using MML within the organ allocation process (Tambur and Das 2023).

In clinical practice, MML computation is always preceded by immunological analyses. Patient’s sera (e.g. from patients enrolled in transplantation programs, pre- and post-transplant) are analysed by Luminex Single Antigen (LSA) beads assay. A LSA assay is a multiplex assay capable of detecting antibody binding to about 200 HLA antigens (among the most frequent ones), fixed on distinct fluorescence beads in a Luminex apparatus. The reactivity patterns are analysed in the light of available knowledge about antibody-verified eplets in order to understand or predict the serum reactivity of a patient with the HLA eplets of a donor (Lhotte 2023). However, inconsistencies sometimes occur between LSA assays and sequence-based MML, raising the need for better eplet and HLA epitope characterization (Tambur and Das 2023).

Several authors have tried to characterize HLA epitopes from a structural point of view in order to refine MML calculation with other properties than AA type. Such properties are for instance, protrusion indices (Ponomarenko *et al.* 2008, Duquesnoy and Marrari 2017, Niemann *et al.* 2024), electrostatic potentials (Mallon *et al.* 2015, 2018), or solvent accessibility (Niemann *et al.* 2022). However, to our knowledge, no study has so far considered HLA epitopes from a dynamic point of view, in particular *via* molecular dynamics (MD) simulations. This is particularly lacking as a recent study suggested that AA flexibility measured along MD trajectories is on average lower on B-cell epitopes than on the rest of the protein surface (Kim *et al.* 2021).

The first interest in MD simulations concerns their ability to refine the 3D structure of proteins, especially to correct stereochemical errors. This technique has proven its usefulness by being successfully integrated into the protocols for predicting the 3D structure of proteins (Mirjalili and Feig 2013, Feig and Mirjalili 2016, Dutagaci *et al.* 2018, Kryshtafovych *et al.* 2019, Heo and Feig 2020) but it has also been used in the refinement of structures obtained experimentally by X-ray diffraction (Burnley *et al.* 2012, Brunger and Adams 2002). Second, MD simulations allow the study of the dynamic properties of proteins, such as side-chain flexibility and solvent accessibility. Finally, MD simulations give access to conformations that are closer to those occurring under ambient conditions. Indeed, the structures obtained in a crystallized conformation are not necessarily the same under ambient conditions. This also applies to predicted structures (Jumper *et al.* 2021), as prediction tools are trained using structures from the Protein Data Bank (https://www.rcsb.org/) (PDB), which mostly consist of X-ray diffraction-resolved structures.

With this in mind, we decided to address the question of HLA epitope characterization from a structural and dynamic point of view, with the goal to better enumerate the potential antibody-binding sites on the surface of HLA antigens. In this article, we report the construction of an unprecedented dataset of high-quality 3D structures for 207 HLA antigens, including those evaluated in LSA assays, and associated with MD simulation records. A set of non-redundant 3D-surface patches, labeled as “Epitope” or “Nonepitope” according to the status of their central AA in HLA Eplet Registry, has been derived from MD trajectories. Descriptors reflecting static and dynamic patch properties have been defined and calculated for all the patches in order to build a dataset to be used with various machine learning (ML) algorithms. Our three best models form the HLA-EpiCheck predictor, which is used to assess the Epitope status of 3D-surface patches corresponding to a subset of unconfirmed eplet positions on HLA DQ antigens. These predictions are compared to experimental validation data and a notable consistency is found. In brief, the aim of this study is to improve, thanks to ML and dynamic 3D structural data, the *in silico* assignment of epitope status to surface patches of HLA antigens in order to contribute to a better matching of donor and recipient during graft allocation.

## 2 Methods

### 2.1 Molecular dynamics data generation

**Figure 2.**
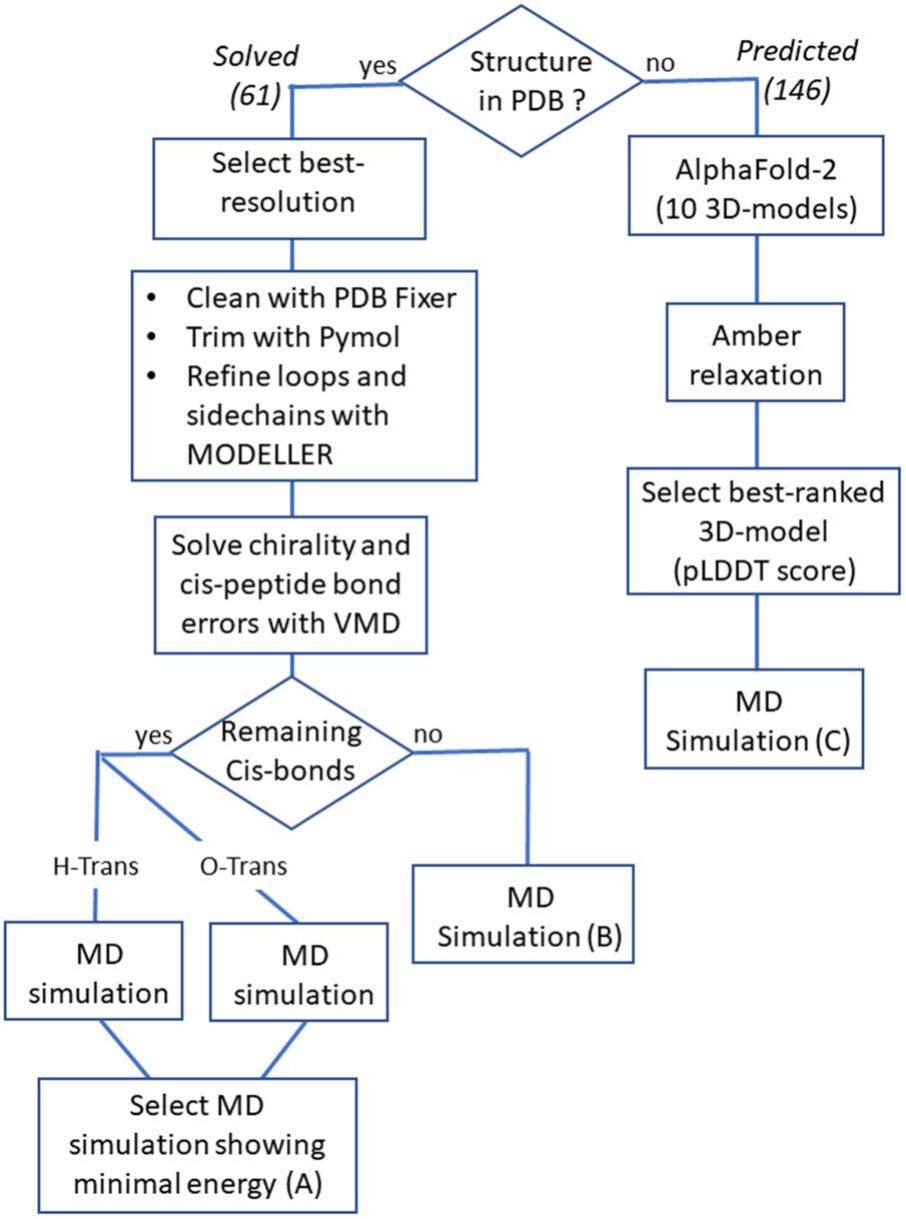
Preparation of the HLA dynamic structural dataset. Left: 61 solved structures including 59 from PDB-REDO. Right: 146 structures predicted using Alphafold-2.

We selected 207 HLA antigens for molecular modeling, which include all the antigens currently tested in LSA flow bead assays (Supplementary Table S1). Two procedures were implemented to generate the refined 3D structures of HLA antigens ready to undergo MD simulations. One procedure corresponds to the antigens for which a structure exists in the PDB and the second one corresponds to antigens lacking a PDB structure (Fig. 2).

#### 2.1. 1 Identification of crystallographic structures in the PDB

The “ncbiblast” API (Altschul *et al.* 1990) was used to find structures in the PDB using HLA sequences from the Immuno Polymorphism Database [IPD-IMGT/HLA database (https://www.ebi.ac.uk/ipd/imgt/hla/index.html)] (Barker *et al.* 2023). Then, we checked if the structure was available in PDB_REDO (https://pdb-redo.eu/about), otherwise, we downloaded the structure from the PDB (collected on 23 June 2020). The resolution values of the structures were obtained from the pdbe/EBI API (https://www.ebi.ac.uk/pdbe/pdbe-rest-api) to finally choose a single structure per antigen according to the best resolution.

PDBFixer (Eastman *et al.* 2017) was used to identify and correct missing atoms or AAs, remove small molecules and groove peptides. Pymol (Schrödinger) was then used to remove signal peptides (if present) and trim the protein to keep only the extracellular globular part (Supplementary Table S2). Loops and side chains were refined with MODELLER (Šali and Blundell 1993). The visual molecular dynamics (VMD) plugins CHIRALITY and CISPEPTIDE (Schreiner *et al.* 2011) were then used to check for chirality and cis-peptide bond errors. In cases where cis-peptide bond errors not involving prolines were identified, structures were generated for hydrogen and oxygen rotations, then MD simulations were performed for both cases and an additional minimization stage was run to choose the conformation that minimized the energy of the system. Otherwise, a single MD simulation was run on the structure (Fig. 2).

#### 2.1. 2 Generation of structures for antigens lacking any structure in the PDB

A local instance of Alphafold v2.1.0 was used to generate the structures of antigens absent from the PDB. In line with the preparation of crystallographic structures, HLA sequences were trimmed to keep only the extracellular globular part (Supplementary Table S2). Default parameters were used for Alphafold-2. Ten 3D models were generated per antigen to which an Amber relaxation was applied (Jumper *et al.* 2021) and a single model was chosen based on the best pLDDT confidence score (Fig. 2).

#### 2.1. 3 Molecular dynamics simulations

MD simulations were performed for each antigen using VMD (Humphrey *et al.* 1996) and the NAMD engine, version 3.0 alpha9 (Phillips *et al.* 2020). To generate the system topology, the VMD AutoPSF tool was used together with the CHARMM36 force field (Lee *et al.* 2016). The system was solvated using the TIP3P explicit solvent model and neutralized using the VMD AutoIonize tool. The Particle Mesh Ewald (PME) method was used to calculate the electrostatic energy with a distance truncation of 11 Å.

The simulations comprised two steps: (i) minimization according to NAMD3 default protocol and (ii) 10 ns MD simulation carried out under NPT conditions, i.e. constant pressure (1 atm) and temperature (310 K). The full set of parameters used for the minimization and MD simulation steps are available in our gitlab repository (https://gitlab.inria.fr/capsid.public_codes/hla-epicheck) in configuration files named “general_min.conf” and “general_MD.conf”. A set of 500 frames from the last 5 ns of simulation (referred to here as MD trajectory) was saved from each MD run for further computation. Short durations were used because as demonstrated elsewhere (Kim *et al.* 2021), side-chain motions are sufficiently fast to be studied through short simulations. We also conducted a posteriori verification showing that dynamic descriptors of 3D-surface patches (see below) computed over longer MD runs (95 ns) exhibit high correlation with those from 5 ns runs (Supplementary Table S10). Additionally, correlation measured for shorter runs (0.5, 1, 2, or 3 ns) compared with the 5 ns run increases with time (Supplementary Table S10). Thus, a 5 ns run duration appears to offer a practical balance, capturing more information than shorter runs while avoiding the higher computational cost of longer ones.

### 2.2 Definition, construction, and labeling of 3D-surface patches

The 3D objects considered in our ML approach are fragments of the protein surface called “patches”. In this study, a 3D-surface patch is centered on a solvent-accessible residue and contains all other solvent-accessible residues present in a radius of 15 Å from the central residue (distances are computed between the centers of mass of the residues). A residue is considered “solvent-accessible” when its average RSASA (relative solvent-accessible surface area) along the trajectory (consisting here of 500 frames) is higher than 20%. The SASA calculator implemented in VMD (Varshney *et al.* 1994) was used to compute the absolute solvent accessibility of residues and the theoretical “ALL” maximum solvent accessibility of each residue defined in Tien *et al.* (2013) (see Supplementary Table S3) was used to calculate RSASA values for each frame. A total of 42 626 solvent-accessible residues was identified from our 207 MD trajectories and all the corresponding 3D-surface patches were computed using in-house written Pymol and Python scripts.

From HLA Eplet Registry, we obtained a list of antibody-verified eplets present in the 207 HLA antigens of this study. A 3D-surface patch is labeled as “Epitope” when its central solvent-accessible residue is part of an eplet from this list. We thus obtained 2117 Epitope patches (Supplementary Table S4). The “Nonepitope” patches are centered on solvent-accessible residues that are not listed as part of an eplet (neither antibody-verified nor unconfirmed) in HLA Eplet Registry. However, not all such patches are eligible as negative counterparts of Epitope patches. A first condition is that, in addition to the central residue, none of the solvent-accessible residues composing a Nonepitope patch should be part of an eplet (either antibody-verified or not). Another condition is that no patch can be composed exclusively of locus-conserved positions. Indeed, locus-conserved positions could be misleading as these positions could by chance display B-cell epitope behavior but they cannot be recognized as such because they cannot raise immune response in humans as they are conserved throughout all individuals. In other words, these locus-conserved positions could bias the training by introducing “positive unlabeled” examples in the Nonepitope class. Using these conditions, we obtained 4769 Nonepitope patches (Supplementary Table S4).

### 2.3 Definition and calculation of 3D-surface patch descriptors

#### 2.3. 1 Static descriptors

Static descriptors (Table 1) are attached to a patch and do not vary along MD simulation. We compute residue hydrophobicity according to the Kyte–Doolittle scale (Kyte and Doolittle 1982) and derive four descriptors for a patch: the hydrophobicity of its central residue, and the minimum, maximum, average residue hydrophobicity over all the residues of the patch.

**Table 1.** Descriptors of 3D-surface patches.^a^

Name	Description (per patch)	Stat.	Dyn.	Value range and unit
H_central	Hydrophobicity^b^ of central residue	x		[−4.5, 4.5]
H_patch_min	Minimum residue hydrophobicity	x		[−4.5, −3.5]
H_patch_max	Maximum residue hydrophobicity	x		[−0.8, 4.5]
H_patch_avg	Average of all residue hydrophobicities	x		[−3.2, 0.3]
Pos_central	Positive charge of central residue	x		{0, 1}
Pos_patch	Sum of positive charges of all residues	x		[0, 11]
Neg_central	Negative charge of central residue	x		{−1, 0}
Neg_patch	Sum of negative charges of all residues	x		[−11, 0]
S_central_min	Minimum RSASA^c^ of central residue over MD		x	[0, 82.6]%
S_central_max	Maximum RSASA of central residue over MD		x	[21.6, 133]%
S_central_avg	Mean RSASA of central residue over MD		x	[10.2, 105.6]%
S_patch_min	Weighted^d^ average of all residues minimum RSASAs over MD		x	[9.2, 30.3]%
S_patch_max	Weighted average of all residues maximum RSASAs over MD		x	[26.4, 62.6]%
S_patch_avg	Weighted average of all residues mean RSASAs over MD		x	[18.8, 43.2]%
F_central	N-RMSF^e^ of central residue		x	[−2, 11.3] Å
F_patch_min	Minimum residue N-RMSF		x	[−2, 0.4] Å
F_patch_max	Maximum residue N-RMSF		x	[−0.2, 17.7] Å
F_patch_Avg	Weighted average of all residues N-RMSFs		x	[−0.91, 1.85] Å

a “Stat.” and “Dyn.” columns refer to the static and dynamic types of descriptors, respectively. The “Value range” corresponds to the values obtained with the NRd dataset.

b Kyte–Doolittle hydrophobicity scale was used, from most hydrophilic (−4.5) to most hydrophobic (+4.5).

c RSASA: relative solvent-accessible surface area.

d Weights (of residues) correspond to the frequency of presence of each residue in the patch over MD (% of frames).

e N-RMSF: normalized-root mean square fluctuation during MD (*z*-score normalization).

The other static descriptors are derived from the residue electrostatic charges, namely a negative charge for aspartate (D) and glutamate (E) residues and a positive charge for lysine (K) and arginine (R) residues. We first describe the charge of the central residue: the attribute “Pos_central” takes the value +1 for a positively charged residue, otherwise 0, and the attribute “Neg_central” takes the value −1 for a negatively charged residue, otherwise 0. Second, the sum of the charges (either positive or negative) of the residues in the patch is calculated and yields a positive integer for the attribute “Pos_patch”, and a negative integer for the attribute “Neg_patch”.

#### 2.3. 2 Dynamic descriptors

Dynamic descriptors (Table 1) measure conformational changes of the patch structure during MD simulation. The first group of six descriptors reflects the variation of RSASA percentage during the run. RSASA values are calculated per residue for each of the 500 frames considered. For the central residue of a patch, we keep the minimum, maximum, and average RSASA values along the run. For the entire patch, we use the minimum, maximum, and average RSASA values of each residue in the patch along the run and we aggregate these values as three weighted averages (over all minimum, maximum, and average values, respectively) in which the weights correspond for each residue to the % of frames in which this residue is really present in the patch during the run (distance to central residue ≤15 Å).

A second group of four descriptors reflects side-chain flexibility along the run. These descriptors are derived from the normalized root mean squared fluctuation (N-RMSF) values computed as described in Kim *et al.* (2021). One corresponds to the N-RMSF of the central residue of a patch and the others to the minimum, maximum, and weighted average N-RMSF over all residues of the patch (weights are the same as defined above). RMSF values are calculated from the 500 frames considered for each trajectory using VMD. The *Z*-score method is used for normalization including all solvent-accessible residues except for five residues at each terminus because of their artefactual flexibility.

### 2.4 Dataset preparation for machine learning

The 207 HLA antigens prepared for this study correspond to 189 distinct allele sequences distributed among HLA loci as detailed in Table 2. Due to the high homology between HLA alleles, the derived dataset composed of the 2117 Epitope and 4769 Nonepitope 3D-surface patches is highly redundant. A “non-redundant” (NRd) dataset was therefore generated by applying the MMseqs2 software (Steinegger and Ding 2017) to the set of 189 HLA sequences with an AA sequence identity threshold of 90%. A total of 17 clusters of HLA sequences was obtained. From each cluster, the HLA sequence corresponding to the antigen with the highest number of Nonepitope patches was kept as representative member in order to maximize collection of Nonepitope patches. Then, Epitope patches were collected from the antigens corresponding to all alleles of the cluster (not only the representative) in order to capture the epitope diversity within each cluster. Redundant Epitope patches (centered on the same eplet residue) were removed.

**Table 2.** Number of modeled antigens per locus in the HLA dynamic structural dataset and percentage of solvent-accessible (surface) AAs computed from 3D static (3D stat.) and MD data.^a^

Locus	3D models (*Seq.*)	Chain (AAs)	% surface AAs	
			3D stat.	MD data
A	34 (*34*)	*α* (276)	51.4	55.4
B	59 (*59*)	*α* (276)	50.3	54.8
C	17 (*17*)	*α* (276)	51.4	56.1
DP	31 (*7 DPA1*	*α* (183)	49.8	53.4
	*and 19 DPB1*)	*β* (191)	52	56.4
DQ	30 (*13 DQA1*	*α* (186)	49.9	52.7
	*and 14 DQB1*)	*β* (192)	51.4	56.1
DR	36 (*29 DRB1*,	*β* (192)	50.2	55.6
	*3 DRB3, 2 DRB4*			
	*and 2 DRB5*)			
Total	207 (*189*)			

a The number of distinct allele sequences (*Seq.*) is shown in parentheses for each locus. Percentages of solvent-accessible AAs have been averaged over all antigens of each locus. Results are shown only for the polymorphic chain identified in the “Chain” column with its length (in AAs) in the 3D model.

The resulting NRd dataset is composed of 243 Epitope and 421 Nonepitope patches and represents about one-tenth of the initial dataset. The distribution of the central residues of these Epitope and Nonepitope patches on the antigen surface is illustrated in Supplementary Fig. S1 for one antigen of each HLA locus. This figure shows that there are no specific hot-spots for either Epitope or Nonepitope patches. To avoid learning bias due to the limited size of the NRd dataset, we performed 10 different random splits respecting the Epitope/Nonepitope ratio and generated 10 pairs of 90% training: 10% test sets (Supplementary Table S5). All performance metrics were computed with appropriate *scikit-learn* scripts.

### 2.5 Model selection for HLA epitope predictor

The following learning algorithms: decision tree (DT; *cart* algorithm), extremely randomized trees (ET), gradient boosted decision trees (GT) were used on the NRd dataset in their *scikit-learn* default implementation. To ensure a robust and reliable model selection process, a combination of grid search and cross-validation techniques using the Python *scikit-learn* library was implemented. For each learning algorithm, *GridSearchCV* was used to explore and tune the hyperparameters using the 10 training sets. The best hyperparameter combination was the one maximizing the average *F*1-score over the 10 training sets, i.e. over 100 × 10 models (10 repetitions of 10-fold cross-validation for each of the 10 training sets). Supplementary Table S6 shows the set of parameters explored in the *GridSearchCV* procedure for each learning algorithm and highlights in bold the best selected combination. For each pair of training/test sets, the performance of the best model trained on the training set was then measured on the test set using *F*1-score.

### 2.6 Validation on unconfirmed eplets

The task of experimental validation of unconfirmed eplets has been undertaken at the Hôpital Saint-Louis, Paris. It involves adsorption experiments of patient’s sera on normal spleen mononuclear cells (SMCs) and DQ-transfected murine cell clones. The results used in this article concern 17 unconfirmed eplets present on HLA DQ antigens for which experimental results have been published recently (Devriese *et al.* 2024).

By definition, eplets can be present on more than one HLA antigen (Fig. 1). To be declared experimentally validated, an eplet must be recognized by one or more antibodies (produced by a human individual and usually tested from a patient’s serum) regardless of the antigen carrying it. Similarly, the prediction concerning a 3D-surface patch centered on an eplet residue for a given antigen should be consistent over all antigens carrying this eplet. We therefore defined aggregated prediction scores at the eplet level. The process is slightly different for single or composite eplets.

A “single” eplet is composed of a unique residue. The aggregation consists of a simple average of individual prediction scores according to (1):

(1) $$S_{r}=\frac{\sum_{a\in A_{r}}pred(r,a)}{\left|A_{r}\right|}$$

where $S_{r}$ is the aggregated score for a unique eplet residue *r* over a set of antigens *A_r_* carrying this eplet, *pred*(*r*, *a*) is the prediction score for the patch centered on residue *r* in antigen *a*.

A “composite” eplet is composed of more than one residue. Here, we aggregate first over all residues of the eplet for each concerned antigen by keeping the maximal prediction score (2) and then we average over all antigens containing this eplet according to (3)

(2) $$P_{a,e}=\max_{r\in R_{a,e}}p\mathrm{red}(r,a)$$

(3) $$S_{e}=\frac{\sum_{a\in A_{e}}P_{a,e}}{\left|A_{e}\right|}$$

Here, $P_{a,e}$ is the prediction score aggregated on $R_{a,e}$, the set of solvent-accessible residues members of a composite eplet *e* for antigen *a*, and *pred*(*r*, *a*) is the prediction score for the patch centered on residue *r* in antigen *a*. Then, $S_{e}$ is the aggregated score for composite eplet *e*, *A_e_* being the set of antigens carrying this eplet.

The two aggregated scores range from 0 to 1 and we consider that our prediction confirms an eplet if the corresponding aggregated score is greater than 0.5.

### 2.7 Summary of HLA-EpiCheck approach

Supplementary Fig. S2 synthesizes our end-to-end approach and represents the complete dataflow of this study.

## 3 Results

### 3.1 HLA epitope and nonepitope 3D-surface patches: a dynamic structural dataset

The main originality of this study is to exploit MD data collected on HLA antigens to enhance the prediction of HLA epitopes. Table 2 describes the unprecedented dynamic structural HLA dataset generated for this purpose. The 207 HLA antigens correspond essentially to the ones used in routine LSA flow bead assays and cover the six major loci of the HLA system (complete list in Supplementary Table S1). The 500 frames corresponding to the last 5 ns of short MD trajectories (10 ns) performed on each antigen have been saved for further computation. To illustrate the interest of using dynamic structural information, we computed the number of solvent-accessible AAs (RSASA ≥20%) for each antigen either on static 3D structure or during MD trajectories. The results, averaged by HLA locus and displayed in Table 2, show that the percentages obtained are systematically higher with MD data than with static data, by about 4%. This indicates that dynamic data may reveal important solvent-accessible AAs (from 5 to 12 depending on the chain length) that are overlooked when using only static data.

The HLA 3D-surface patches (15 Å radius) considered in this study are centered on a solvent-accessible residue (with solvent accessibility defined dynamically along MD run). Thus, each 3D-surface patch is identified by its central residue (nature and position, *α* or *β* chain) and by the HLA antigen structure from which it was computed. For HLA epitope prediction, HLA patches are labeled as Epitope or Nonepitope according to the classification of their central residue in HLA Eplet Registry (see Section 2). From the 207 HLA antigens considered in this study we computed a total of 42 626 3D-surface patches of which 2117 were labeled as Epitope and 4769 as Nonepitope (redundant Rd dataset). Amino acid composition, solvent accessibility, and side-chain flexibility were analysed for Epitope and Nonepitope patches. Results presented in Supplementary Table S7 and Fig. S3 indicate that a complex set of significant differences exists between the two classes of patches, further motivating our goal of building a HLA epitope prediction system.

### 3.2 Model selection for HLA epitope prediction

**Figure 3.**
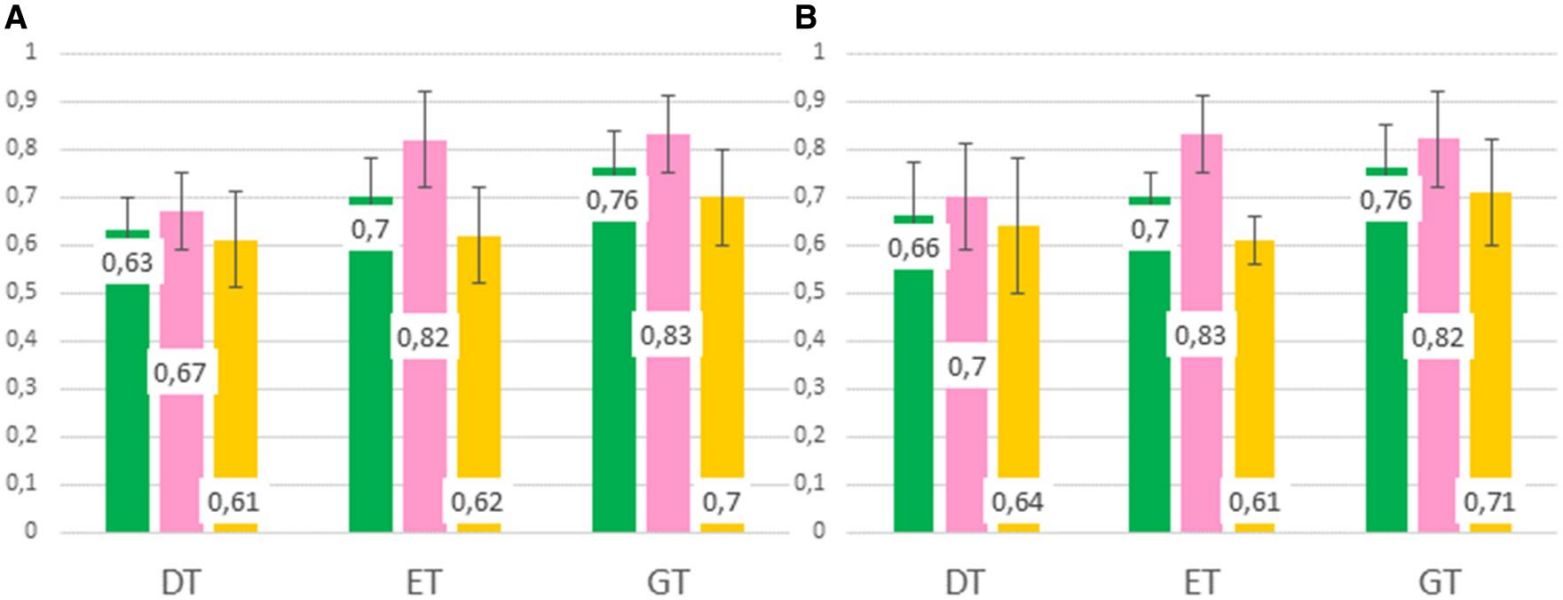
Performance metrics for the three tree-based classifiers (DT: decision tree, ET: extra trees, GT: gradient trees) on the NRd dataset. *F*1-score (green), precision (pink), and recall (yellow) are displayed in (A) as average values over the 10 training sets (10 repetitions of 10-fold cross-validation each) and in (B) as average values over the 10 test sets. Following good machine-learning practices, samples of each test set were evaluated using the model built on the training set obtained from the same train-test split. All calculations were performed with the Epitope label taken as the positive class. All values and standard deviations are reported in Supplementary Table S8.

After redundancy reduction (see Section 2) we obtained the NRd dataset composed of 243 Epitope and 421 Nonepitope patches. For prediction purpose, eight static and 10 dynamic descriptors were defined (Table 1) and computed for each 3D-surface patch (see Section 2). Intensive model selection was performed on the NRd dataset for three tree-based classifiers: DT, ET, and GT. The performance metrics obtained under optimal hyperparameters conditions (Supplementary Table S6) on both training and test sets are displayed in Fig. 3.

The *F*1-scores obtained on the training sets (panel A) increase from 0.63 to 0.70 and 0.76 between simple DT and more sophisticated tree-based models (ET, GT). In all three cases, the precision value is greater than the recall value, indicating a greater capacity of the model to discriminate between Epitope and Nonepitope patches than to retrieve all Epitope patches from the dataset. In other words, the three systems generate less false positive than false negative predictions. Nevertheless, the differences between precision and recall are not so big, revealing well-balanced models. Moreover, the values obtained by the three models on the 10 test sets (panel B) are very consistent with those obtained on the training set, indicating the absence of overfitting.

Given the relatively close performances obtained with the three algorithms, we decided to continue with the three of them. We built representative models on the complete NRd dataset and studied feature importance in each model.

### 3.3 Feature importance for HLA epitope prediction

**Figure 4.**
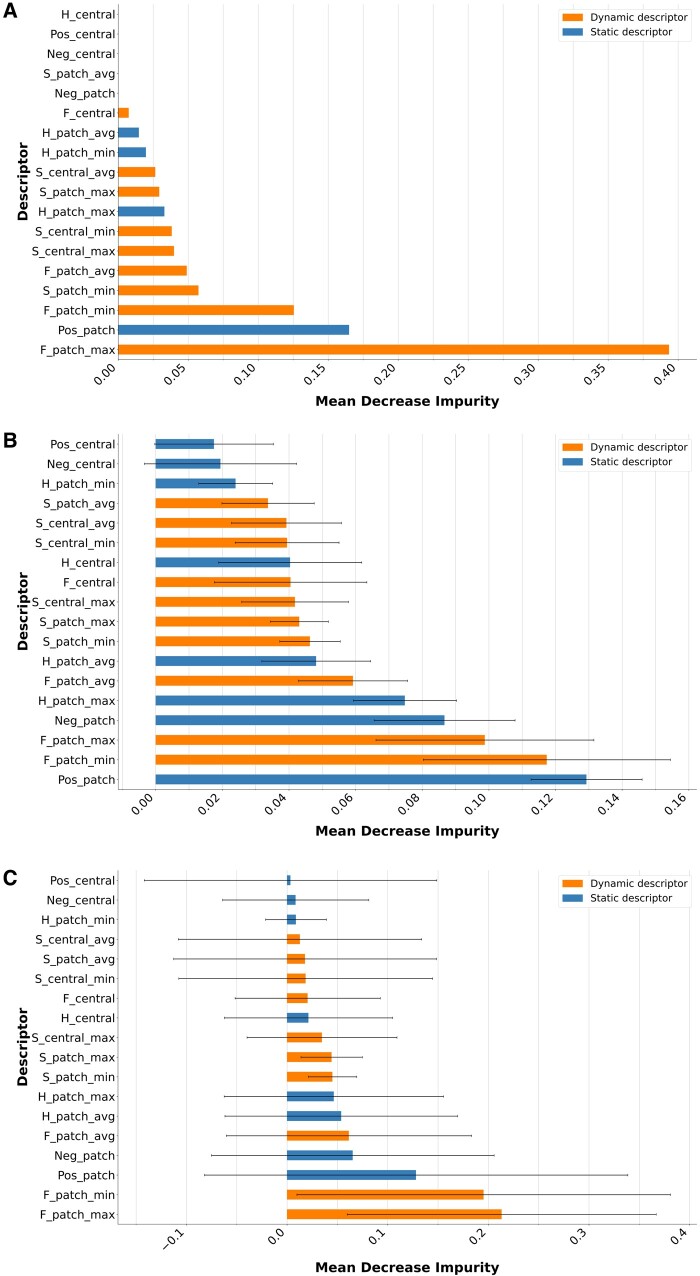
MDI feature importance using *F*1-metric for DT (A), ET (B), and GT (C) models. Horizontal bar length corresponds to MDI values. Whiskers correspond to standard deviations of MDI values over all the trees of the model (ET and GT). Orange bars correspond to dynamic descriptors and blue bars to static ones.

To evaluate the contribution of dynamic and static descriptors to the performance of our three models, we applied the mean decrease in impurity (MDI) method proposed by *scikit-learn*. In brief, this method computes for each feature the total reduction in impurity (calculated as the Gini index) contributed by all splits in a tree involving that feature. The measure is then averaged over all the trees of the model (for ET and GT). This method is computationally very efficient and has been widely used in a variety of applications despite some known biases involving scale variability between features and high cardinality of categorical features. In our dataset, there is no categorical feature and the value ranges of all our descriptors do not differ more than one order of magnitude (Table 1). The results present in Fig. 4 are normalized MDI values for each descriptor with the sum of MDIs for all descriptors being equal to 1.

It appears that dynamic descriptors (orange bars) contribute significantly to all three models. The sum of their contribution is equal to 0.767, 0.560, and 0.664, i.e. greater that the contribution of static descriptors for DT, ET, and GT models, respectively. For DT, the most important contribution is provided by the F_patch_max feature (0.394) and largely overcomes all other features. This feature represents the maximal side-chain flexibility (N-RMSF) over all residues present in the 3D-surface patch. For ET, the best contributor is a static one (Pos_patch) corresponding to the sum of the positive charges present in a 3D-surface patch, but the two flexibility features F_patch_max and F_patch_min rank 2nd and 3rd, respectively. In the case of GT model, the interpretation is difficult due to the enormous standard deviation calculated over all trees in the model. This is probably due to the fact that trees are smaller and likely display larger heterogeneity in GT than in ET model. Nevertheless, the three best-ranked features remain the same two flexibility features (that contribute to the model for about 0.4), and the Pos_patch feature also found at 1st rank in ET and 2nd rank in DT. In addition to highlighting the importance of dynamic features for Epitope recognition, this analysis also stresses the importance of considering descriptors at the patch rather than residue level to capture global characteristics necessary for Epitope recognition.

**Figure 5.**
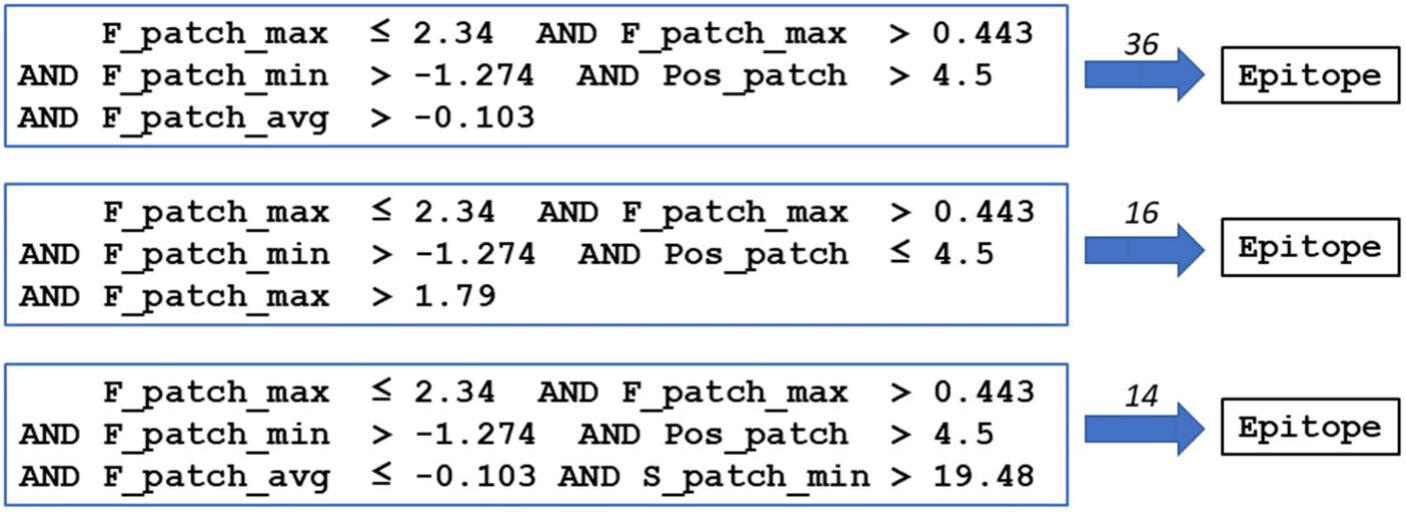
Classification rules extracted from the global decision tree built with the whole NRd dataset. The rules correspond to the paths leading to the three most populated pure epitope leaves (see also Supplementary Fig. S4).

We then studied the DT model as this type of model can be visualized and interpreted through explicit rules. The full model is shown in Supplementary Fig. S4. It has a total of 81 nodes, a maximal depth of 11 and among the 41 leaves, none has less than three samples. All pure Epitope leaves (blue color, circled in red) contain more than 10 samples each, including one with 36 samples. In total, 100 Epitope samples are classified in pure Epitope leaves (about 37% of the 272 Epitope samples in the dataset). The classification rules obtained for the three most populated pure Epitope leaves are listed in Fig. 5. Most of the rule items involve descriptors associated with side-chain flexibility at the level of the patch (F_patch_max, _min and _avg) including the root criterion stating that the maximal F_patch value should not exceed 2.34. This is in line with the current knowledge that side-chain flexibility is in average lower in B-cell epitopes than in non-epitope regions (Kim *et al.* 2021). We actually reproduced this finding with our Rd dataset of Epitope and Nonepitope 3D-surface patches (Supplementary Fig. S3). Furthermore, all items of the three rules in Fig. 5 involve “patch” rather than “central” residue (eplet) properties, further underlining the relevance of using patches to represent epitope candidates.

The complexity of the two other models was also analysed. The number of trees, total number of nodes, average number of nodes per tree, average depth of the trees, and number of leaves are reported in Table 3. The most efficient model (GT) contains twice as many trees as ET but the trees look less complex in terms of all other characteristics.

**Table 3.** Complexity of the three HLA-EpiCheck models.

Characteristic	DT	ET	GT
Nb of trees	1	500	1000
Total Nb of nodes	81	155 814	13 480
Average Nb of nodes per tree	81	311.6	13.5
Average depth of trees	11	17.4	3.0
Nb of leaves	41	78 157	7240

### 3.4 Comparison with experimental results on a subset of unconfirmed eplets

HLA-EpiCheck prediction was applied to the set of 24 unconfirmed DQ eplets from HLA Eplet Registry that have been experimentally validated in our recently published study (Devriese *et al.* 2024). These unconfirmed eplets are absent from our initial dataset but they are present on a certain number of HLA antigens from our HLA dynamic structural dataset. Only 17 from these 24 eplets have at least one solvent-accessible residue available for 3D-surface patch construction. The corresponding patches (one per eplet residue and per concerned HLA antigen for a total of 369) were retrieved from the 42 626 3D-surface patches pre-computed from the 207 HLA antigens. No specific trend in the localization of these patches was observed (Supplementary Fig. S5). The dynamic and static descriptors were computed and prediction obtained with the three models for each 3D-surface patch. Individual patch prediction scores were then aggregated to compute the $S_{r}$ and $S_{e}$ scores for single and composite eplets, respectively, as described in Section 2. Table 4 summarizes the results obtained with each of the three models. With a threshold arbitrarily set to 0.50, we count the highest number of validated eplets (12/17) with the DT model, although the least efficient regarding *F*1-score. The ET and GT models predict nine and eight validated eplets, respectively. Consistency between the three models predictions and with experimental validation concerns at most eight eplets. In view of the variability of the scores obtained with the three models for each given eplet, we hypothesize that each model has its own advantages and we propose to keep all three of them as part of a prediction system named HLA-EpiCheck.

**Table 4.** HLA-EpiCheck scores for 17 unconfirmed HLA DQ eplets experimentally validated.^a^

		$S_{r}$ or $S_{e}$ scores				
No	Eplet	DT	ET	GT	Chain	Nb.
1	70GT	**0.92**	**0.79**	**1**	*β*	4
2	66DR	**0.82**	**0.84**	**1**	*β*	8
3	67VG	**0.63**	**0.80**	**1**	*β*	7
4	40ERV	**0.76**	**0.60**	**0.94**	*α*	15
5	74EL	**0.77**	**0.76**	**0.91**	*β*	18
6	66ER	**0.80**	**0.73**	**0.91**	*β*	14
7	185I	**0.67**	**0.56**	**0.67**	*β*	11
8	75I^b^	**0.55**	**0.55**	**0.66**	*α*	26^c^
	161DI^b^	0.32	0.32	0.23	*α*	26^c^
9	129QS	**0.74**	0.28	0.46	*α*	8
10	130R	**0.50**	**0.60**	0.38	*β*	29
11	135D	**0.52**	0.33	0.37	*β*	30
12	125G	**0.50**	0.00	0.36	*β*	2
13	167R	0.20	0.36	0.30	*β*	25
14	129H	0.34	0.36	0.30	*α*	24
15	3P	0.40	0.41	0.00	*β*	2
16	130A	0.37	0.16	0.00	*α*	3
17	23L	0.04	0.34	0.00	*β*	2

a Scores for the three HLA-EpiCheck models are reported and highlighted in bold when greater or equal to 0.50. Ranking is according to the GT score. The concerned chain of the antigen structure (Chain) and the number of antigens (Nb.) carrying the eplet are indicated. Rows 1-8 are consistent Epitope predictions across the three models and rows 9-12 are inconsistent predictions with only one or two scores ≥0.5.

b Eplet 75I is divided into two eplets here as it is composed of two groups of AA distant of nearly 50 Å from each other.

c This set of antigens comprises alleles families DQA1*02, DQA1*04, DQA1*05, and DQA1*06 that carry a deletion relative to the DQA1*01 and DQA1*03 allele families at position 56. The residue numbering indicated in the table corresponds to the non-deleted alleles. The homologous 3D patches were considered (despite the different position number) when computing the average scores.

The five eplets with low prediction scores (less than 0.5 with all three models) were further investigated. In particular, the HLA-EpiCheck scores relative to neighboring solvent-accessible residues were checked. In the case of eplet 23 L, several patches centered on neighboring residues were predicted as HLA epitopes suggesting that a HLA epitope could be present in the close vicinity of eplet 23 L. Unfortunately, for the other four low-score eplets, no similar explanation could be found so far.

## 4 Discussion

Our novel approach for predicting HLA epitopes is based on a unique HLA dynamic structural dataset that includes refined 3D structures (solved and predicted) of 207 HLA antigens and their associated MD simulations. This has allowed us to compute 3D-surface patches and describe them with dynamic descriptors. In contrast with pHLA3D-3.0, the recent database of 3D structures of HLA molecules (Teles e Oliveira *et al.* 2021), we used AlphaFold-2 prediction rather than homology modeling for the 146 HLA antigens absent from the PDB and our 3D structures lack the immunogenic peptide to avoid confusion between B-cell and T-cell epitopes.

To our knowledge, it is the first time that 3D-surface patches combined with dynamic descriptors derived from MD trajectories are used in a ML context. Dynamic descriptors not only concern side-chain flexibility but also solvent accessibility and we show that during MD about 4% additional AAs become exposed on the surface (Table 2). Thus, important structural properties of a protein may remain overlooked when dealing only with static 3D structures. An alternative to the use of MD simulations for taking into account protein flexibility in ML studies could be the sampling of variant conformations using Rosetta Relax tool (https://docs.rosettacommons.org/docs/latest/application_documentation/structure_prediction/relax) (Conway *et al.* 2014). However, this should be the focus of future work as it will require testing the computational cost of the Relax method compared with short MD simulations and its suitability for capturing variations in amino acid solvent accessibility and side-chain flexibility.

We have evaluated the contribution of static and dynamic types of patch descriptors to HLA epitope prediction through MDI feature importance analysis. The three best-ranked features for the three models analysed in this study are two dynamic descriptors related to side-chain flexibility in a patch and one static descriptor related to positive charges in a patch. This is consistent with the recent report that side-chain flexibility is key in antigen–antibody recognition (Kim *et al.* 2021) and with current knowledge about the role of electrostatic interactions in antigen–antibody complexes, in particular for HLA epitopes (Kosmoliaptsis *et al.* 2011, Mallon *et al.* 2015, 2018, Gu *et al.* 2019). However, these types of descriptors, reflecting the dynamics of antigen surfaces and aggregated at the patch level, are not often found in general-purpose B-cell epitope predictors. In this active field of ML, most effort is devoted to encoding AAs known to interact with antibodies. The most performant tool today is DiscoTope-3.0 (Høie *et al.* 2024) that exploits inverse folding representation of AAs and ensemble XGBoost models trained on <1500 structures. However, we found that this efficient general-purpose tool does not perform well with HLA epitope prediction task (Supplementary Table S8). This may be due to the fact that HLA antigens are poorly represented in the structural training set used by DiscoTope-3.0 as this training set actually covers the widest possible variety of antigens. Reciprocally, we anticipate that HLA-EpiCheck would likely show poor performance when tested on B-cell epitopes other than HLA epitopes. The failure of DiscoTope-3.0 on HLA epitope prediction fully justifies our efforts to develop a specific predictor dedicated to HLA epitopes.

Indeed, HLA epitope prediction is a particularly difficult task as it takes place in a highly redundant context. The size of our initial set of 3D-surface patches drastically decreased from 6886 to 664 patches only after reducing sequence redundancy at a 90% cutoff. We tried to take advantage of the more numerous redundant dataset using the K-nearest neighbor (KNN) algorithm which is an instance-based predictor, working in a transductive manner by inferring the class of a tested sample on the basis of the class of the three most similar samples. The KNN method is preferred in situations where reducing redundancy before the training phase is too costly a task. Assessment of KNN prediction (*k* = 3) using the Rd dataset yielded higher *F*1-scores (0.81 by cross-validation on the training set and 0.84 on the test set) than the three models trained on the NRd dataset. However, the KNN predictor did not outperform HLA-EpiCheck in identifying experimentally validated eplets (Supplementary Table S9; 11 instead of 12 validated eplets).

A limitation of this study lies in the inherent variability of MD simulations. While we verified on a few randomly selected antigens that equilibrium was reached after 5 ns simulation, the dynamic descriptors computed from different replicates displayed varying correlation levels depending on the replicate pair (Supplementary Table S11). Future work should examine the impact of MD stochasticity on ML outcomes, as this is an emerging area of investigation. Another limitation of HLA-EpiCHeck is the performance of its three components (*F*1-score not higher than 0.76). This low but honest baseline may be improved in future in particular for reducing the amount of false negative. The NRd dataset will be updated with new reliable samples labeled as Epitope based on the ongoing assessment of “antibody-verified” eplets from HLA Eplet registry (Bezstarosti *et al.* 2021, Tambur and Das 2023). New descriptors based on surface geometry and further exploiting MD trajectories can be added in order to capture HLA epitope specific features. Moreover, surface patch encoding via embedding methods as described in the MaSIF approach could be explored (Gainza *et al.* 2020).

The complexity of organ allocation task makes it difficult to determine what type of MML calculation would be the most appropriate to prevent DSA formation and enhance graft survival. Although several studies have reported that eplet MML between recipient and donor could be correlated with DSA formation (Wiebe *et al.* 2019, Sapir-Pichhadze *et al.* 2020, Senev *et al.* 2020), there is still today a great heterogeneity in the concepts and methods for MML calculation and evaluation (Tambur and Das 2023). Interestingly, a ML prediction score, based on NetMHCII system, has already been proposed for recipient–donor mismatch assessment regarding predicted differences in T-cell epitopes (Lachmann *et al.* 2017). Although this approach presented several limitations (Guidicelli *et al.* 2018, Lachmann *et al.* 2018), it suggests that *in silico* predictions could contribute to MML calculation. In a similar frame of mind, we propose that HLA-EpiCheck scores, assigned to eplets that are not yet antibody-verified, could be included in MML methods.

Future work should also consider the complementarity (or lack thereof) of 3D surface patches to recipient’s antibodies. This would require acquisition of structural information on 3D complexes between donor HLA antigens and recipient antibodies. For the moment, the PDB repository only contains one such complex that was experimentally solved (PDB: 9b7b). However, more structural data may soon be available for donor–recipient pairs in which both donor HLA antigens and recipient antibodies have been typed and sequenced, as the 3D structure of the complexes could be predicted by AlphaFold.

## 5 Conclusion

We introduce HLA-EpiCheck, a HLA epitope prediction system composed of three tree-based models trained on 3D structures and MD simulations of 207 HLA antigens. Feature importance analysis confirms the relevance of using 3D-surface patches and dynamic descriptors derived from MD trajectories for HLA epitope prediction. Despite its limited global efficiency, HLA-EpiCheck could predict HLA epitopes for 12 of 17 unconfirmed eplets of DQ antigens recently validated experimentally. Retrospective clinical studies are needed to demonstrate the possible contribution of HLA-EpiCheck predictions to donor–recipient HLA matching when allocating transplants. More generally, we hope that this work will encourage the use of dynamic structural descriptors in conjunction with static AA representations for any other ML task involving protein 3D structure.

## Supplementary Material

vbae186_Supplementary_Data

## Data Availability

Structural data and MD trajectories are deposited as open data under doi:10.57745/GXZHH8. In-house scripts and machine-learning models for HLA-EpiCheck are available from https://gitlab.inria.fr/capsid.public codes/hla-epicheck.
