# Supplementary material for: HLA-EpiCheck: novel approach for HLA B-cell epitope prediction using 3D-surface patch descriptors derived from molecular dynamic simulations
Source: Bioinform Adv. 2024 Dec 5;4(1):vbae186. doi: 10.1093/bioadv/vbae186 (PMC11631505; doi:10.1093/bioadv/vbae186)
Supplement: vbae186_Supplementary_Data [file vbae186_supplementary_data.pdf]

Bioinformatics Advances, 2024, 1–2
doi: DOI HERE
Advance Access Publication Date: Day Month Year
Paper

HLA-EpiCheck: novel approach for HLA B-cell epitope prediction using 3D-surface patch descriptors derived from molecular dynamic simulations

Diego Amaya-Ramirez,[1,*] Magali Devriese,[2] Romain Lhotte,[2] Cédric Usureau,[2] Malika Smaïl-Tabbone,[1] Jean-Luc Taupin[2] and Marie-Dominique Devignes[1]

*Corresponding author. diego.amaya-ramirez@loria.fr

Abstract

This appendix contains supplementary tables and figures that will be available online (address provided by the journal).
See main paper for full abstract.
Note that Tables and Figures spanning over two columns are positioned at the top of a page. This introduces unwanted page breaks.

1. SUPPLEMENTARY TABLES

The list of supplementary Tables is as follows.

Table S1 presents the full list of modeled HLA antigens.

Table S2 presents the length of modeled sequences per locus. Only the extracellular globular part of the protein was modeled.

Table S3 presents the maximal solvent accessible surface per amino acid in a polypeptide chain. This is the reference for calculating the RSASA values.

Table S4 presents the composition of the initial redundant dataset (6886 3D-surface patches).

Table S5 presents the composition of the nonredundant dataset after sequence redundancy reduction (664 3D-surface patches).

Table S6 presents the list of hyperparameters used in gridsearch for optimizing the three tree-based ML models.

Table S7 compares the AA distribution and RSASA per AA type in the two sets of AAs derived from Epitope and Nonepitope patches. Significant differences are highlighted.

Table S8 presents the performance metrics of the various ML algorithms tested on training and test sets. In A., the DT, ET and GT models have been trained on the NRd dataset as described in the main paper. The values obtained are represented as a bar diagram in Figure 2 of the main paper. In B. KNN (k=3) was used on Rd dataset. ET was trained and tested on Rd training (80%) and test (20%) sets ; DiscoTope-3.0 was tested on the Rd test set.

Table S9 presents the prediction scores obtained for the 17 unconfirmed eplets experimentally validated with KNN (using the Rd dataset), and ET-Rd (Extra-Trees model trained on Rd dataset).

Table S10 presents the correlation of the dynamic descriptors values between various pairs of MD simulation lengths.

Table S11 presents the correlation of the dynamic descriptors values between pairs of MD replicates for 5ns MD simulations.

© The Author 2024. Published by Oxford University Press. All rights reserved. For permissions, please e-mail: journals.permissions@oup.com

2. SUPPLEMENTARY FIGURES

The list of supplementary Figures is as follows.

Figure S1 illustrates the mapping of all patch centers of the NRd dataset onto 6 HLA 3D structures (one per locus) for Epitope and Nonepitope patches.

Figure S2 schematizes the dataflow along the HLA-EpiCheck approach.

Figure S3 shows the cumulative probability curves for AA N_RMSF values in Epitope versus Nonepitope patches.

Figure S4 shows the decision tree obtained from the whole non-redundant dataset.

Figure S5 shows the positions of eplet residues tested by HLA-EpiCheck in Table 4 on a HLA DQ 3D structure.

Table S1. List of modeled HLA antigens. DP and DQ antigens are composed of two polymorphic protein chains, produced from the DPA1 and DPB1, and DQA1 and DQB1 loci, respectively.

Locus	Antigens
A	A*01:01, A*02:01, A*02:02, A*02:03, A*02:05, A*02:06, A*02:07, A*03:01, A*11:01, A*11:02, A*23:01, A*24:02, A*24:03, A*25:01, A*26:01, A*29:01, A*29:02, A*30:01, A*30:02, A*30:03, A*31:01, A*32:01, A*33:01, A*33:03, A*34:01, A*36:01, A*43:01, A*66:01, A*66:02, A*68:01, A*68:02, A*69:01, A*74:01, A*80:01
B	B*07:02, B*08:01, B*13:01, B*13:02, B*14:01, B*14:02, B*15:01, B*15:02, B*15:03, B*15:10, B*15:11, B*15:12, B*15:13, B*15:16, B*18:01, B*27:03, B*27:04, B*27:05, B*27:06, B*27:08, B*27:09, B*35:01, B*35:08, B*37:01, B*38:01, B*39:01, B*40:01, B*40:02, B*40:06, B*41:01, B*41:03, B*41:04, B*42:01, B*42:02, B*44:02, B*44:03, B*44:05, B*45:01, B*46:01, B*47:01, B*48:01, B*49:01, B*50:01, B*51:01, B*51:02, B*52:01, B*53:01, B*54:01, B*55:01, B*56:01, B*57:01, B*57:03, B*58:01, B*59:01, B*67:01, B*73:01, B*78:01, B*81:01, B*82:01
C	C*01:02, C*02:02, C*03:02, C*03:03, C*03:04, C*04:01, C*05:01, C*06:02, C*07:02, C*08:01, C*08:02, C*12:03, C*14:02, C*15:02, C*16:01, C*17:01, C*18:02
DP	DPA1*01:03-DPB1*01:01, DPA1*01:03-DPB1*02:01, DPA1*01:03-DPB1*03:01, DPA1*01:03-DPB1*04:01, DPA1*01:03-DPB1*04:02, DPA1*01:03-DPB1*06:01, DPA1*01:03-DPB1*11:01, DPA1*01:03-DPB1*19:01, DPA1*01:03-DPB1*23:01, DPA1*01:03-DPB1*28:01, DPA1*01:04-DPB1*18:01, DPA1*01:05-DPB1*03:01, DPA1*01:05-DPB1*18:01, DPA1*01:05-DPB1*28:01, DPA1*02:01-DPB1*01:01, DPA1*02:01-DPB1*03:01, DPA1*02:01-DPB1*05:01, DPA1*02:01-DPB1*06:01, DPA1*02:01-DPB1*09:01, DPA1*02:01-DPB1*13:01, DPA1*02:01-DPB1*14:01, DPA1*02:01-DPB1*15:01, DPA1*02:01-DPB1*17:01, DPA1*02:01-DPB1*18:01, DPA1*02:02-DPB1*05:01, DPA1*02:02-DPB1*10:01, DPA1*02:02-DPB1*11:01, DPA1*02:02-DPB1*13:01, DPA1*03:01-DPB1*13:01, DPA1*03:01-DPB1*20:01, DPA1*04:01-DPB1*28:01
DQ	DQA1*01:01-DQB1*05:01, DQA1*01:01-DQB1*06:02, DQA1*01:02-DQB1*05:01, DQA1*01:02-DQB1*05:02, DQA1*01:02-DQB1*06:02, DQA1*01:02-DQB1*06:04, DQA1*01:02-DQB1*06:09, DQA1*01:03-DQB1*06:01, DQA1*01:03-DQB1*06:03, DQA1*02:01-DQB1*02:01, DQA1*02:01-DQB1*02:02, DQA1*02:01-DQB1*03:01, DQA1*02:01-DQB1*03:02, DQA1*02:01-DQB1*03:03, DQA1*02:01-DQB1*04:01, DQA1*02:01-DQB1*04:02, DQA1*03:01-DQB1*02:01, DQA1*03:01-DQB1*03:01, DQA1*03:01-DQB1*03:02, DQA1*03:01-DQB1*03:03, DQA1*03:02-DQB1*03:02, DQA1*03:02-DQB1*03:03, DQA1*03:03-DQB1*04:01, DQA1*04:01-DQB1*02:01, DQA1*04:01-DQB1*04:02, DQA1*05:01-DQB1*02:01, DQA1*05:03-DQB1*03:01, DQA1*05:05-DQB1*03:01, DQA1*05:08-DQB1*02:01, DQA1*06:01-DQB1*03:01
DR	DRB1*01:01, DRB1*01:02, DRB1*01:03, DRB1*03:01, DRB1*03:02, DRB1*04:01, DRB1*04:02, DRB1*04:03, DRB1*04:04, DRB1*04:05, DRB1*07:01, DRB1*08:01, DRB1*09:01, DRB1*09:02, DRB1*10:01, DRB1*11:01, DRB1*11:04, DRB1*12:01, DRB1*12:02, DRB1*13:01, DRB1*13:03, DRB1*14:01, DRB1*14:02, DRB1*14:54, DRB1*15:01, DRB1*15:02, DRB1*15:03, DRB1*16:01, DRB1*16:02, DRB3*01:01, DRB3*02:02, DRB3*03:01, DRB4*01:01, DRB4*01:03, DRB5*01:01, DRB5*02:02

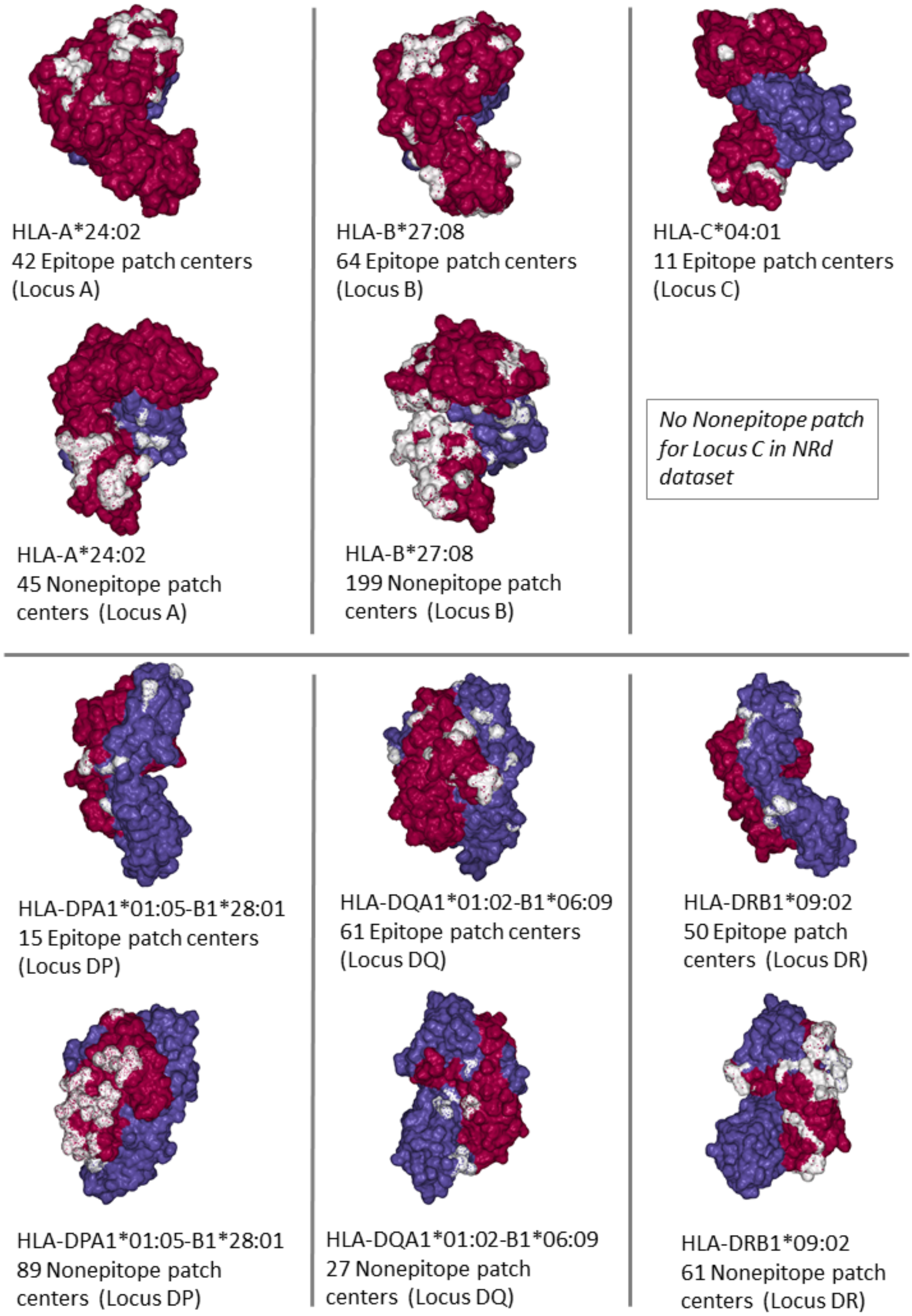

Fig. S1: Screenshots of 3D representations of HLA antigens with patch centers from the NRd dataset highlighted in white. Chain A is colored in red and β-microglobulin (locus A,B and C) or chain B (locus DP, DQ, DR) are colored in blue. Clickable links to original 3D representation stored using 3dRS (3D structure Representation Sharing) service at IRB (Institute for Research in Biomedicine) Barcelona are as follows: HLA-A*24:02 (42 Epitope patch centers) ; HLA-A*24:02 (45 Nonepitope patch centers) ; HLA-B*27:08 (64 Epitope patch centers) ; HLA-B*27:08 (199 Nonepitope patch centers) ; HLA-C*04:01 (11 Epitope patch centers) ; HLA-DPA1*01:05-DPB1*28:01 (15 Epitope patch centers) ; HLA-DPA1*01:05-DPB1*28:01 (89 Nonepitope patch centers) ; HLA-DQA1*01:02-DQB1*06:09 (61 Epitope patch centers) ; HLA-DQA1*01:02-DQB1*06:09 (27 Nonepitope patch centers) ; HLA-DRB1*09:02 (50 Epitope patch centers) ; HLA-DRB1*09:02 (61 Nonepitope patch centers).

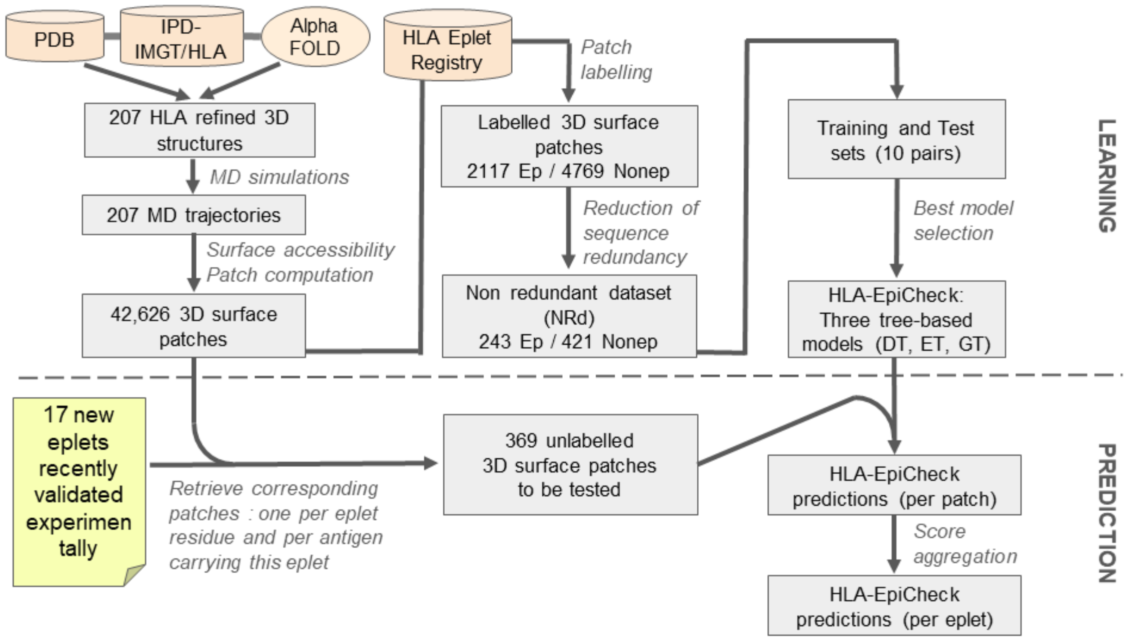

Fig. S2: Schematization of the dataflow along the HLA-EpiCheck approach. The top part corresponds to the "LEARNING" step that includes data preparation, training and testing and results in three prediction models. The bottom part corresponds to the "PREDICTION" step (or use-case) in which a set of eplets, with status "unconfirmed" in HLA Eplet Registry but recently validated experimentally by us, is submitted to prediction by HLA-EpiCheck. MD: Molecular Dynamics, Ep : Epitope label, Nonep : Nonepitope label, NRd : Non Redundant, DT : Decision Tree, ET : ExtraTrees, GT GradientTrees.

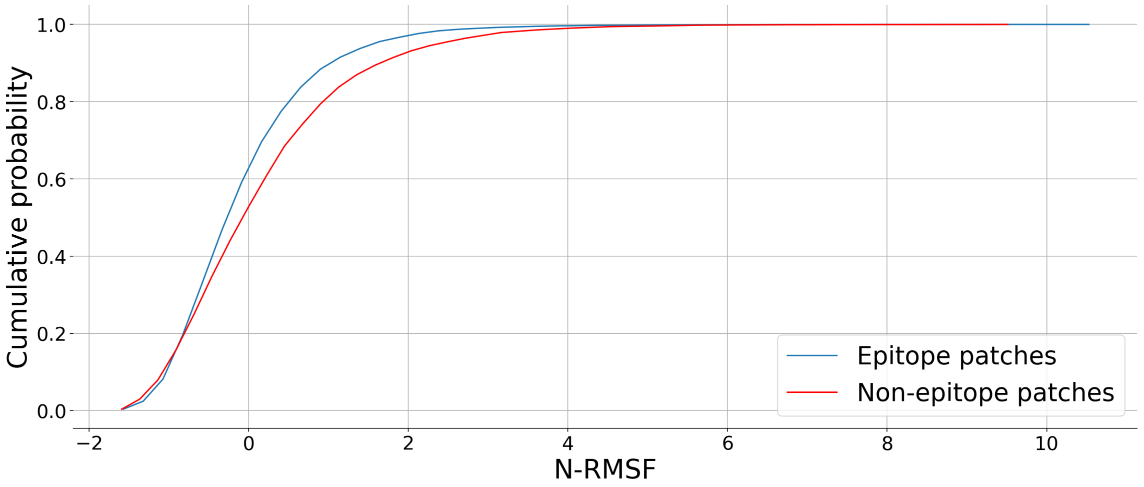

Fig. S3: Cumulative probability of N_RMSF values for all AAs members of Epitope patches (blue curve ; 36,545 values) versus Nonepitope patches (red curve ; 84,107 values). Kolmogorov-Smirnov statistical analysis yielded a p-value of 1.1×10^{-267} for the difference between the two curves.

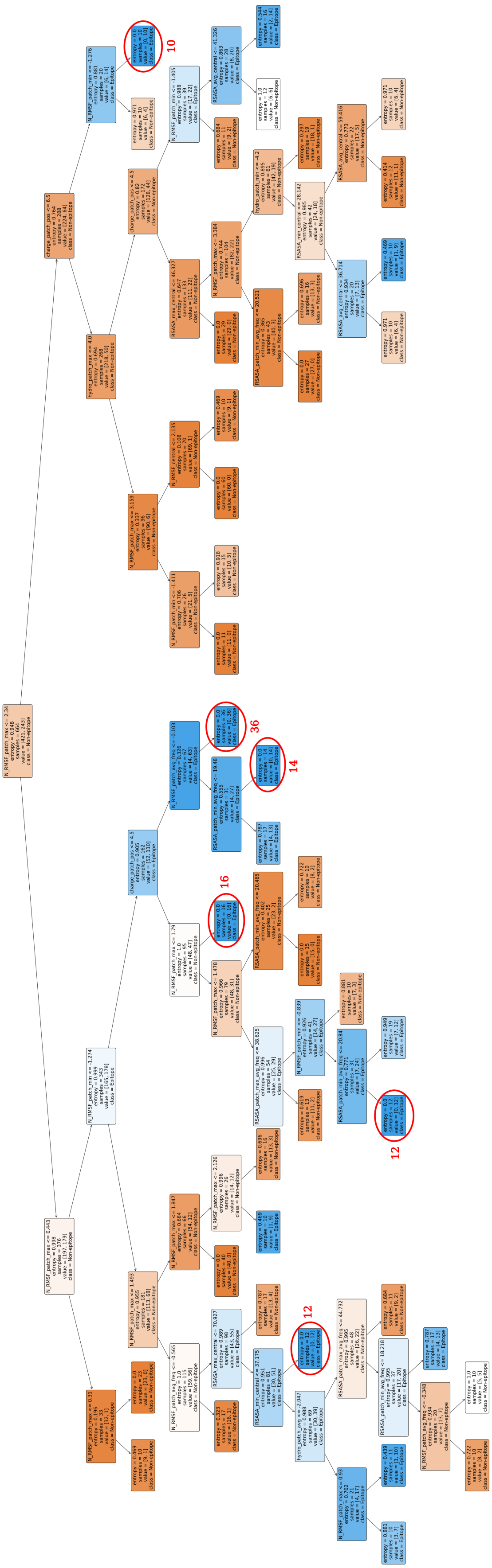

Fig. S4: Decision tree built on the whole NRd dataset. The *scikit-learn* implementation of *cart* algorithm was used. Nodes and leaves with a majority of Epitope samples are colored in blue. Pure leaves with more than 10 Epitope samples are circled in red and the number of samples is indicated.

Table S2. Length of modeled sequences per locus. Only the extracellular globular part of the protein was modeled, so the signal peptide, the amino acids linking the extracellular globular part to the transmembrane part, the transmembrane part and the intracellular part were excluded.

locus	chain	length in 3D model
**A, B, C**	**chain α**	276
	**β2-microglobulin**	99
**DP**	**chain α**	183
	**chain β**	191
**DQ**	**chain α**	186
	**chain β**	192
**DR**	**chain α**	182
	**chain β**	192

Table S3. Maximum solvent accessibility of residues (Tien et al., 2013)

Residue	Max. accessibility Å^2
ALA	138
ARG	285
ASN	204
ASP	204
CYS	169
GLU	233
GLN	234
GLY	114
HIS	231
ILE	208
LEU	211
LYS	246
MET	227
PHE	251
PRO	166
SER	161
THR	182
TRP	295
TYR	274
VAL	184

Table S4. Overview of the redundant ML dataset. In the training (80%) and test (20%) sets, samples (3D patches) are stratified according to the class: Epitope (Epi.) versus Nonepitope (Nonepi.) and the gene loci (A, B, C, DP, DQ and DR) encoding the HLA antigens they belong to.

		Dataset		Training set		Test set	
		**Epi.**	**Nonepi.**	**Epi.**	**Nonepi.**	**Epi.**	**Nonepi.**
**# samples**	**A**	434	656	353	506	81	150
	**B**	529	2822	427	2270	102	5527
	**C**	177	359	134	281	43	78
	**DP**	184	637	138	529	46	108
	**DQ**	486	107	395	95	91	12
	**DR**	307	188	241	141	66	47
	**Total**	2117	4769	1688	3822	429	947
**# antigens**	**A**	34		27		7	
	**B**	59		47		12	
	**C**	17		13		4	
	**DP**	31		24		7	
	**DQ**	30		24		6	
	**DR**	36		28		8	
	**Total**	207		163		44	

Table S5. Overview of the NRd dataset and the ten derived pairs of training (90%) / test (10%) sets. Samples (3D patches) are stratified according to the class: Epitope (Epi.) versus Nonepitope (Nonepi.). Training and test set counts are shown as the mean $\pm$ standard deviation of the 10 values corresponding to the 10 pairs of training/test sets. The antigen distribution could not be preserved between training and test sets because the number of samples is too small. In particular, we observed that all alleles derived from gene C are clustered by SeqsMM-2 with alleles derived from gene B. The representative member of this cluster being a B allele, this lead to the absence of any Nonepitope samples corresponding to gene C.

	Dataset		Training set		Test set	
	**Epi.**	**Nonepi.**	**Epi.**	**Nonepi.**	**Epi.**	**Nonepi.**
**A**	42	45	37.8±1.8	40.7±1.1	4.2±1.8	4.3±1.1
**B**	64	199	56.1±3.5	177.9±3.1	7.1±3.5	21.1±3.1
**C**	11	0	10.2±0.9	0	0.8±0.9	0
**DP**	15	89	12.8±0.9	80.3±2.4	2.2±1.0	8.8±2.4
**DQ**	61	27	55.0±2.0	24.4±1.7	6.0±2.0	2.6±1.6
**DR**	50	61	45.3±1.9	54.80±1.9	4.7±1.9	6.2±1.9
**Total**	243	421	218	378	25	43

Table S6. Hyperparameters used in the gridsearch procedure . The best values found are underscored and in bold.

Learning algorithm	Hyperparameters used in gridsearch
Decision Tree	criterion: "gini", **'entropy'**, 'log_loss' ; max_features: **None**, 'sqrt', 'log2' ; min_samples_leaf: **1**, 2, 3, 5, 10 ; min_samples_split: **2**, 3, 5, 10 ; splitter: **'best'**, 'random'
Extra Trees	criterion: **"gini"**, 'entropy', 'log_loss' ; max_features: **None**, 'sqrt', 'log2' ; min_samples_leaf: **1**,2,3,4 ; min_samples_split: 2,**3**,4,5 ; n_estimators: 50, 100, 150, 200, 250, **300**, 400, 500, 600, 700, 900,1000 ; warm_start: **False**, True.
Gradient Trees	criterion: **'friedman_mse'**, 'squared_error' ; learning_rate: 0, 0.2, 0.3, **0.5**, 0.7 ; loss: 'log_loss', **'exponential'** ; max_features: **None**, 'sqrt', 'log2' ; min_samples_leaf: **1**, 2, 3 ; min_samples_split: 2, 3, 5, 7, **10**, 15 ; n_estimators: 100, 200, 300, 400, 400, 500, 600, 700, 800, 900, 1000, 1200, **1400**, 1600, 1700 ; subsample: 0.2, 0.5, 0.7, 0.8, **1** ; warm_start: True, **False**.

Table S7. Differences between HLA Epitope and Nonepitope patches. The two sets of AAs (Epitope 36,545 AAs ; Nonepitope 84,107 AAs) contained all surface AAs present in the 2117 and 4769 Epitope and Nonepitope 3D-surface patches, respectively (Rd dataset). AA types are grouped according to their physicochemical properties. The two columns under header "AA (%)" display the frequency of occurrence of AAs (in %) in each set of AA derived from Epitope (Ep.) and Nonepitope patches (NonE). Statistical Chi^2 test performed at `https://biostatgv.sentiweb.fr/?module=tests/chideux` provides a p-value estimated at 0, revealing that the AA distribution in the two sets is significantly different. The four columns under header "Med. RSASA (%)" display for each set of AA derived from Epitope and Nonepitope patches the number of AAs of a given type and the median RSASA value measured over MD trajectories of all these AAs. Differences in median RSASA greater than 5% are indicated in bold (the numbers of considered AAs strongly support the fact that most observed differences are significant). "NPNH" = Non Polar and Non Hydrophobic ; "NPH" = Non Polar and Hydrophobic ; "PUC" = Polar uncharged ; "PC" = Polar charged ; "NA" = Non Applicable.

		**AA %**		**Med. RSASA (%)**			
**Group**	**AA**	**Ep.**	**NonE.**	**Ep**		**NonE.**	
				Nb	RSASA	*Nb*	RSASA
NPNH	GLY	4.9	4.7	*1791*	**34.3**	*3953*	**43.9**
	PRO	5.2	6.2	*1900*	46.5	*5215*	44
NPH	ALA	5.4	6.6	*1973*	**42.2**	*5551*	**34.7**
	VAL	5.1	5.4	*1864*	31.9	*4542*	35.9
	LEU	3.4	2.6	*1243*	30.3	*2187*	31.2
	ILE	2.6	1.1	*950*	**39.1**	*925*	**44.4**
	MET	0.4	0.4	*146*	28.9	*336*	26.3
	PHE	1.2	0.7	*439*	**55**	*589*	**37.9**
	TRP	1.8	1.0	*658*	25.7	*841*	23.2
	CYS	0.1	0	*37*	26.9	*0*	NA
PUC	SER	6.0	7.0	*2193*	37	*5887*	36.1
	THR	6.1	9.4	*2229*	**41.2**	*7906*	**35.7**
	TYR	4.6	3.2	*1681*	29.1	*2691*	23.8
	ASN	3.5	2.6	*1279*	38.0	*2187*	34.9
	GLN	8.0	10.0	*2924*	41.2	*8411*	38.1
	HSD	3.0	1.0	*1096*	**43.8**	*841*	**35.5**
PC	ASP	6.9	9.7	*2522*	38.6	*8158*	39.5
	GLU	12.6	11.2	*4605*	**41.7**	*9420*	**49.2**
	LYS	4.7	4.5	*1718*	**50.7**	*3785*	**55.7**
	ARG	14.4	12.1	*5262*	39.9	*10177*	35.9

Table S8. Performance metrics for the various tools explored in this study on the non-redundant (A.) and redundant (B.) datasets. A. Values obtained for the training and test sets with the NRd dataset are the average ± standard-deviation over the 10 pairs of training and test sets. B. For the Rd dataset, the value obtained for the training set (80% of Rd dataset) is the average ± standard-deviation over the 10 repetitions of 10-fold cross-validation, and a single value is obtained for the test set (20% of Rd dataset). DT, ET and GT are for Decision Tree, ExtraTrees and GradientTrees, respectively. KNN is for K-Nearest Neighbour with K=3. For comparison purpose, we also tested DiscoTope-3.0 on the Rd test set. The program was run on the 207 HLA antigens considered in this study and the results were filtered on the central residue of the 3D patches present in the test set.

		A. Non redundant	
Algo.	Metrics	Training	Test
DT	F1	0.63 ± 0.07	0.66 ± 0.11
	Prec.	0.67 ± 0.08	0.7 ± 0.11
	Recall	0.61 ± 0.10	0.64 ± 0.14
ET	F1	0.70 ± 0.01	0.70 ± 0.05
	Prec.	0.82 ± 0.02	0.83 ± 0.08
	Recall	0.62 ± 0.01	0.61 ± 0.05
GT	F1	0.76 ± 0.08	0.76 ± 0.09
	Prec.	0.83 ± 0.08	0.82 ± 0.10
	Recall	0.70 ± 0.10	0.71 ± 0.11
		B. Redundant	
Algo.	Metrics	Training	Test
KNN	F1	0.81 ± 0.02	0.84
	Prec.	0.85 ± 0.03	0.92
	Recall	0.77 ± 0.03	0.77
ET	F1	0.88 ± 0.02	0.87
	Prec.	0.92 ± 0.02	0.93
	Recall	0.83 ± 0.03	0.82
DiscoT.	F1	-	0.55
	Prec.	-	0.56
	Recall	-	0.55

Table S9. Predictions obtained for 17 unconfirmed HLA DQ eplets experimentally validated using KNN or ET-Rd. KNN (k=3) was used with the Rd dataset as a source of labelled examples. Normalisation of descriptor values and Euclidean distance were used to compute distances. ET-Rd was trained on Rd dataset as explained in the caption of Table S8. Eplets are ordered as in Table 4 to facilitate comparisons.

		$\mathcal{S}_r$ or $\mathcal{S}_e$ scores			
**No**	**Eplet**	**KNN**	**ET-Rd**	**Chain**	**Nb.**
1	70GT	**1**	**0.80**	β	4
2	66DR	**0.95**	**0.95**	β	8
3	67VG	**0.94**	**0.78**	β	7
4	40ERV	**0.81**	**0.98**	α	15
5	74EL	**0.94**	**0.99**	β	18
6	66ER	**0.92**	**0.76**	β	14
7	185I	0.27	**0.66**	β	11
8	75I*	**0.85**	**0.66**	α	27**
	161DI*	0.47	0.42	α	27**
9	129QS	**0.91**	**0.53**	α	8
10	130R	0.11	0.36	β	29
11	135D	0.43	0.40	β	30
12	125G	0.33	**0.60**	β	2
13	167R	0.33	0.34	β	25
14	129H	**0.77**	**0.53**	α	24
15	3P	0.00	0.4	β	2
16	130A	**0.50**	0.28	α	3
17	23L	0.33	0.47	β	3

*: Eplet 75I is divided in two eplets here as it is composed of two groups of AAs distant of nearly 50Å from each other.

**: This set of antigens comprises alleles families DQA1*02, DQA1*04, DQA1*05 and DQA1*06 that carry a deletion relative to the DQA1*01 and DQA1*03 allele families at position 56. The residue numbering indicated in the table corresponds to the non deleted alleles. The homologous 3D patches were considered (despite the different position number) when computing the average scores.

Table S10. Influence of MD run duration on dynamic descriptor values. In this study, three replicates of MD simulation were conducted on six HLA antigens (listed in Figure S1) for a duration of 100ns. From the six antigens, we derived a subset of 241 3D-surface patches (Epitope and Nonepitope) from our NRd dataset. Dynamic descriptors were calculated for various frame sets (10ps interval), representing different MD simulation lengths: frames 501-550 (0.5ns), 501 to 600 (1ns), 501-700 (2ns), 501-800 (3ns), 501-1000 (5ns) and 501-10,000 (95ns, in blue). For each replicate and descriptor, Pearson correlation coefficients were calculated between the 241 values obtained at these lengths and those at length 5ns, as shown in the column headers. Correlation gradually increases between 0.1ns and 5ns, indicating that descriptors calculated from shorter runs are not entirely equivalent to those from 5ns runs. Correlation coefficients between the 95ns and 5ns lengths are above 0.7 for all 10 dynamic descriptors, suggesting that a 5ns duration balances comprehensiveness with computational cost. Other studies, such as Kim et al. (2021), similarly employ 5ns MD runs to investigate side chain flexibility in epitopes.

**Length vs 5ns**	**0.5ns**	**1ns**	**2ns**	**3ns**	**95ns**
**Descriptor**					
**Rep.1**					
F_central	0.999	0.999	1	1	0.990
F_patch_min	0.974	0.978	0.986	0.993	0.963
F_patch_max	0.904	0.932	0.942	0.974	0.861
F_patch_avg	0.993	0.995	0.996	0.998	0.988
S_central_min	0.939	0.953	0.968	0.983	0.882
S_central_max	0.903	0.924	0.956	0.975	0.854
S_central_avg	0.942	0.960	0.978	0.989	0.896
S_patch_min	0.840	0.875	0.923	0.964	0.799
S_patch_max	0.736	0.808	0.891	0.939	0.723
S_patch_avg	0.806	0.858	0.918	0.956	0.762
**Rep.2**					
F_central	0.999	1	1	1	0.996
F_patch_min	0.989	0.992	0.995	0.997	0.986
F_patch_max	0.921	0.940	0.955	0.976	0.855
F_patch_avg	0.994	0.996	0.998	0.999	0.982
S_central_min	0.938	0.960	0.975	0.988	0.887
S_central_max	0.911	0.938	0.963	0.980	0.844
S_central_avg	0.945	0.960	0.981	0.992	0.889
S_patch_min	0.820	0.885	0.937	0.965	0.758
S_patch_max	0.749	0.829	0.902	0.944	0.679
S_patch_avg	0.789	0.856	0.921	0.954	0.722
**Rep.3**					
F_central	1	1	1	1	0.992
F_patch_min	0.973	0.980	0.990	0.992	0.954
F_patch_max	0.952	0.959	0.971	0.987	0.795
F_patch_avg	0.994	0.996	0.998	0.999	0.972
S_central_min	0.941	0.955	0.977	0.986	0.887
S_central_max	0.891	0.917	0.951	0.971	0.833
S_central_avg	0.937	0.953	0.976	0.988	0.892
S_patch_min	0.829	0.881	0.936	0.967	0.756
S_patch_max	0.707	0.785	0.892	0.946	0.706
S_patch_avg	0.767	0.831	0.911	0.957	0.730

Table S11. Pearson correlation coefficients for the 10 dynamic descriptors between pairs of MD replicates (see column headers). Details of the replicates are provided in the caption of Table S10. Correlations were calculated for 5ns MD simulations, with the number of descriptors displaying correlation coefficient > 0.7 indicated in the last row of the table.

**Descriptor**	**Rep.1 vs Rep.2**	**Rep.2 vs Rep.3**	**Rep.3 vs Rep.1**
F_central	0.594	0.714	0.711
F_patch_min	0.208	0.583	0.644
F_patch_max	0.708	0.723	0.576
F_patch_avg	0.406	0.704	0.579
S_central_min	0.916	0.915	0.916
S_central_max	0.905	0.896	0.894
S_central_avg	0.913	0.925	0.921
S_patch_min	0.783	0.776	0.761
S_patch_max	0.702	0.730	0.684
S_patch_avg	0.729	0.739	0.710
$Corr > 0.7$	**7**	**9**	**6**

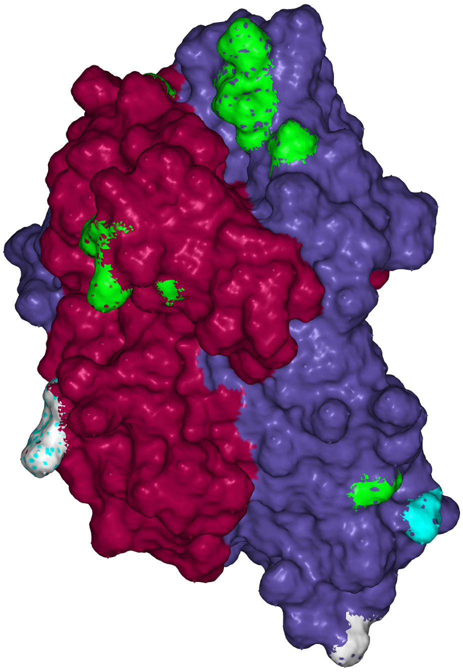

Fig. S5: Screenshot of 3D representation of HLA DQ antigen (HLA-DQA1*0102-DQB1*06.09) with mapped patch centers corresponding to the AAs related to the 17 eplets validated experimentally and submitted to HLA-EpiCheck prediction (see Results, Table 4). Chain A is colored in red and chain B in blue. AAs corresponding to eplets predicted as Epitope by the 3 HLA-EpiCheck models are colored in green (8 eplets, 15 AAs in total), AAs corresponding to eplets predicted as Epitope by at least one HLA-EpiCheck model are colored in cyan (4 eplets, 5 AAs in total). Other AAs not predicted as epitope by HLA-EpiCHeck remain white (5 eplets, 7 AAs in total). Click here to access the original 3dRS representation at IRB Barcelona.
